# Morpho-molecular characterization of *Gyrodactylus* parasites of farmed tilapia and their spillover to native fishes in Mexico

**DOI:** 10.1038/s41598-021-93472-6

**Published:** 2021-07-06

**Authors:** Adriana García-Vásquez, Carlos Daniel Pinacho-Pinacho, Ismael Guzmán-Valdivieso, Miguel Calixto-Rojas, Miguel Rubio-Godoy

**Affiliations:** 1 Instituto de Ecología, A.C., Red de Biología Evolutiva, Carretera antigua a Coatepec 351, 91073 Xalapa, Veracruz Mexico; 2 Investigador Cátedras CONACyT, Instituto de Ecología, A.C., Red de Estudios Moleculares Avanzados, Carretera antigua a Coatepec 351, 91073 Xalapa, Veracruz Mexico

**Keywords:** Ecological epidemiology, Invasive species

## Abstract

Translocation of fishes for aquaculture has resulted in the co-introduction of some of their parasites. African cichlid fishes, generically called “tilapias” have been introduced worldwide, along with their monogenean parasites. In a nation-wide survey, we characterised monogeneans of the genus *Gyrodactylus* infecting farmed “tilapia” throughout Mexico. We also collected native fishes around farms, to look for potential parasite spillover from cultured fishes. Monogeneans were identified taxonomically using morphological and molecular characters. Originally African, pathogenic *Gyrodactylus cichlidarum* was recorded in every farm surveyed, infecting different “tilapia” varieties, as well as three native cichlid fish species. Previously, we had shown that *G*. *cichlidarum* also infects native, non-cichlid fishes in Mexico. We also recorded that *Gyrodactylus yacatli* is widely distributed in Mexico, infecting cultured “tilapia” and native fishes; and present data indicating that this is a further translocated African parasite. A third, unidentified gyrodactylid infected farmed and native fishes in Chiapas, southern Mexico; we describe the new species as *Gyrodactylus shinni* n. sp., and provide evidence that this is a third monogenean translocated with African fish. The wide distribution of exotic parasites co-introduced with “tilapia” and their spillover to native fishes may have an important impact on the ichthyofauna in Mexico, one the world’s megadiverse countries.

## Introduction

Among teleost fishes, the family Cichlidae possesses one of the highest species richness (> 1700 known species), including at least 900 freshwater African species^1^. Monogenean parasites infecting cichlid fishes are similarly diverse; for instance, tropheine cichlids of Lake Tanganyika, which underwent a rapid radiation process are infected by an equally -if not more- diverse assemblage of parasites from the genus *Cichlidogyrus*, which diverged synchronously with their hosts^2^. Starting in the middle of the twentieth century, various cichlids from the genera *Coptodon*, *Sarotherodon* and *Oreochromis* referred to generically as “tilapia” were exported globally from Africa, mainly for aquacultural purposes^1^. Nile tilapia *Oreochromis niloticus* has been introduced throughout the Americas^3,4^ and various other “tilapias” have likewise been translocated worldwide. Several monogenean parasites have been co-introduced along with their fish hosts. Thus, African species of the genera *Cichlidogyrus*^5,6^ and *Gyrodactylus*^7^ have been documented infecting translocated “tilapias” worldwide.

In Mexico, the first recorded introduction of “tilapias” dates from 1945, when redbelly tilapia *Coptodon zillii* was imported from the USA; while in 1964, Nile tilapia was introduced from “Africa” (no further details available) and Costa Rica, and both blue tilapia *O. aureus* and Mozambique tilapia *O. mossambicus* were introduced from the USA; redbreast tilapia *C. rendalli* was imported from Cuba in 1968; and Wami tilapia *O. urolepis* was translocated from Costa Rica in 1978^8^. The first records of introduced monogeneans infecting farmed African “tilapia” in Mexico date from the 1980’s^8^, and by the early 2000’s, evidence of the transfer of African parasites to native, American cichlids became available with the record of *Cichlidogyrus longicornis*, *C. sclerosus* and *C. tilapiae* infecting Lana cichlid *Vieja fenestrata*, and of *Enterogyrus malmbergi* infecting San Domingo cichlid *Thorichthys callolepis*^9^. *Cichlidogyrus sclerosus* has also been recorded from Mayan cichlid *Mayaheros urophthalmus*^10^. *Gyrodactylus cichlidarum* is an originally African parasite that has been translocated worldwide and shown to negatively affect the survival of farmed fishes, particularly juveniles^7,11^. In Mexico, *G. cichlidarum* has been recorded in fish farms throughout the country^12^, from the Yucatán peninsula in the southeast^13,14^ to the northwestern state of Sinaloa^15,16^, as well as the central states of Veracruz^15,16^ and Morelos^17^. *Gyrodactylus yacatli* was previously recorded to infect “tilapia” in southeastern Mexico^7^, and *O. niloticus* and the cichlid *Pseudocrenilabrus philander* in Zimbabwe^18^; but the low prevalence of this species both in Mexico and Zimbabwe, and the unavailability of molecular data for African samples precluded support for any hypothesis on the biogeographical origin of the taxon. Two introduced monogeneans, *C. sclerosus* and *G. cichlidarum*, are considered to have established in Mexico^8^; and both have been recorded infecting native Mexican fishes other than cichlids, as *C. sclerosus* parasitizes endemic blackfin goodea *Goodea atripinnis*^8^ and *G. cichlidarum* infects three native poeciliids: shortfin molly *Poecilia mexicana*, porthole livebearer *Poeciliopsis gracilis* and two spot livebearer *Pseudoxiphophorus bimaculatus*^19^.

**Figure 1:**
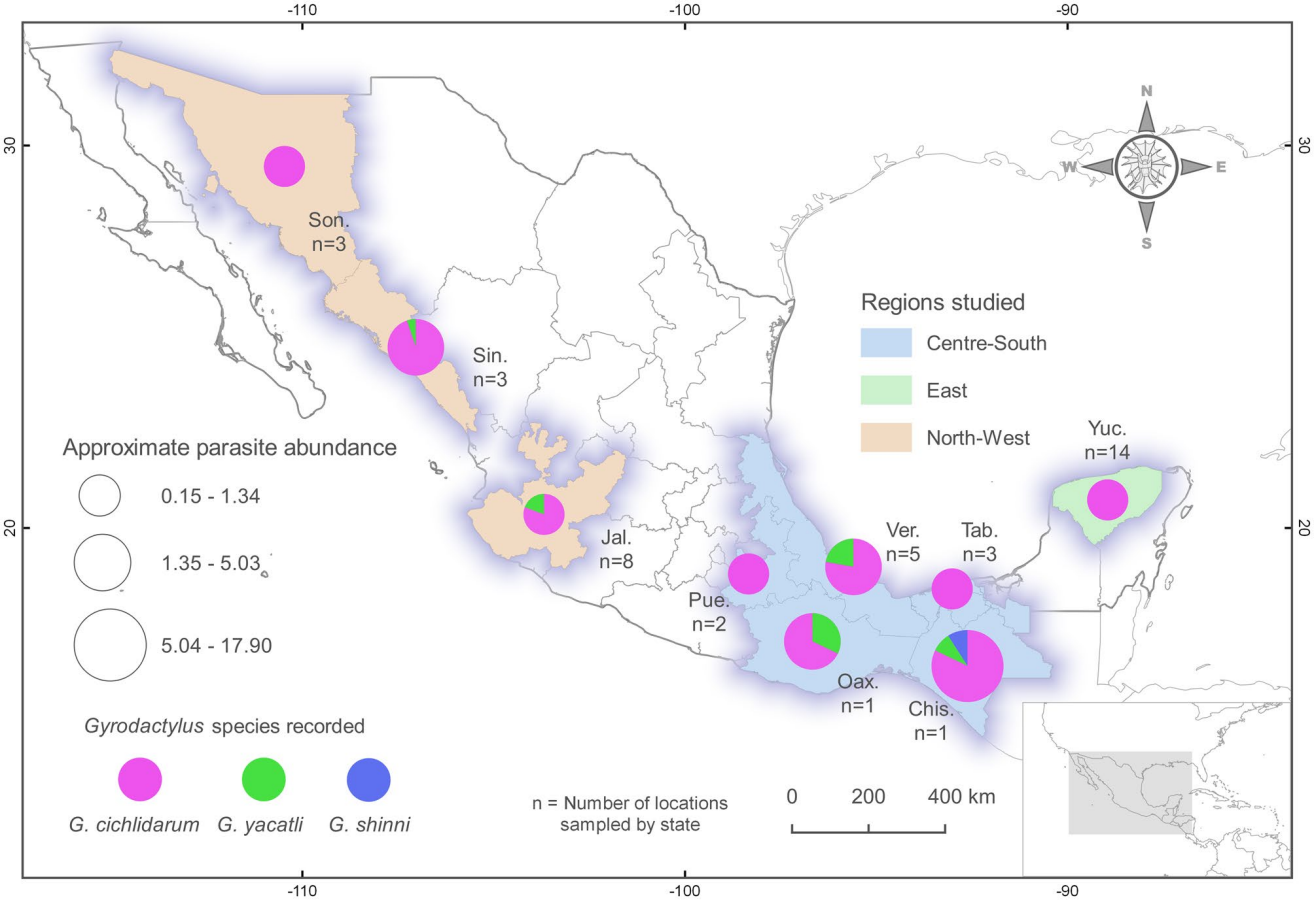
Map of Mexico, showing the distribution of *Gyrodactylus cichlidarum*, *G. yacatli* and *G. shinni* n. sp. and the localities of farms where “tilapia” and native fish were sampled. State names are as follows: Son: Sonora; Sin: Sinaloa; Jal: Jalisco; Pue: Puebla; Ver: Veracruz; Tab: Tabasco; Oax: Oaxaca; Chis: Chiapas; Yuc: Yucatán. We used QGIS version 3.6.2 (http://www.qgis.org) to generate the map. The layers of political division of Mexico were obtained from the CONABIO geoportal (http://www.conabio.gob.mx/informacion/gis/).

In this study, we present the results of an exploratory parasitological survey conducted at some of the most productive “tilapia” farms in Mexico, from the Yucatán peninsula through to Sonora, on the USA border (Fig. 1). The main objectives of this survey were (1) to explore the presence and distribution of the known *Gyrodactylus* species infecting cultured “tilapia” throughout Mexico; (2) to assess the potential spillover of parasites to native fish species in the vicinity of aquaculture facilities, and conversely, look for evidence of host switches from native cichlids to farmed “tilapia”; (3) to better understand the likely biogeographical origin of *G. yacatli*; and (4) to generate morphological and molecular data to explore variation within gyrodactylids infecting “tilapia” throughout Mexico; and, if present, characterize new taxa.

## Results

A total of 40 “tilapia” farms were sampled in three regions in Mexico: North-West (states of Jalisco, Sinaloa and Sonora: n = 14), Centre-South (states of Puebla, Oaxaca, Veracruz, Tabasco and Chiapas: n = 12), and East (state of Yucatán: n = 14) (Fig. 1, Table 1). Approximately 25 healthy “tilapia” individuals were randomly sampled from each farm, sacrificed and stored in 95% ethanol. Native fishes were randomly captured in streams and rivers close to aquaculture facilities in the Centre-South region, including Poeciliidae (*Poecilia formosa*, *Poecilia mexicana*, *Poeciliopsis gracilis*, *Pseudoxiphophorus bimaculatus* and *Xiphophorus hellerii*), Goodeidae (*Goodea atripinnis*), and Profundulidae (*Profundulus oaxacae*). In addition, specimens of *V. fenestrata*, Papaloapan cichlid *Paraneetroplus nebuliferus* and an unidentified native cichlid were collected in Chiapas.

**Table 1:** An overview of the cichlid fishes examined from “tilapia” farms in Mexico where *Oreochromis niloticus* (different strains) and native fish were sampled, and from where gyrodactylids were collected and identified taxonomically.

State	Host	Total worms	Worms processed	*Gyrodactylus* sp.	GenBank acc. no	CNHE acc. no
**1.Centre-South**						
Chiapas (Chis.)	*O. niloticus*	679	36	*Gyrodactylus cichlidarum*	KY489687	11389‡
					KY489688	11389‡
					KY489689	11389‡
					KY489692	11389‡
					KY489693	11389‡
				*Gyrodactylus yacatli*	KY489740	11418**
					KY489741	11418**
					KY489742	11418**
					MN759049	11418**
				*Gyrodactylus shinni* n. sp*-*	MN759066	11387*
					MN759067	11388†
	Unidentified cichlid	37	19	*Gyrodactylus cichlidarum*	–	11390‡
				*Gyrodactylus yacatli*	–	11419**
				*Gyrodactylus shinni* n. sp-	–	11388†
Oaxaca (Oax.)	*Oreochromis niloticus*	139	16	*Gyrodactylus yacatli*	MN759055	11421**
					MN759051	11421**
					MN759056	11421**
					MN759054	11421**
	*Paraneetroplus nebuliferus*	5	2	*Gyrodactylus cichlidarum*	MN759061	11407‡
					MN759060	11407‡
	*Vieja fenestrata*	10	10	*Gyrodactylus cichlidarum*	MN759057	11408‡
					MN759064	11408‡
					MN759065	11408‡
					MN759058	11408‡
					MN759059	11408‡
					MN759062	11408‡
					MN759063	11408‡
				*Gyrodactylus yacatli*	MN759053	11422**
					MN759050	11422**
Puebla (Pue.)	*Oreochromis niloticus*	32	6	*Gyrodactylus cichlidarum*	–	11406**
Tabasco (Tab.)	*Oreochromis niloticus*	47	16	*Gyrodactylus cichlidarum*	KY489686	11401‡
					KY489684	11402‡
					KY489685	11402‡
					KY489691	11402‡
					KY489697	11402‡
					KY489698	11402‡
Veracruz (Ver.)	*Oreochromis niloticus*	130	24	*Gyrodactylus cichlidarum*	–	11416‡, 11417‡
	Florida tilapia	5	3	*Gyrodactylus cichlidarum*	–	11414**
				*Gyrodactylus yacatli*	KY489752	11423**
	Pargo UNAM	390	19	*Gyrodactylus cichlidarum*	–	11411‡, 11412‡, 11413‡
				*Gyrodactylus yacatli*	–	11425**
	Rocky Mountain	50	17	*Gyrodactylus cichlidarum*	–	11410‡
				*Gyrodactylus yacatli*	KY489745	11424**
					KY489746	11424**
					KY489747, MN759052	11424**
					KY489748	11424**
					KY489749	11424**
					KY489750	11424**
					KY489756	11424**
**2. North-West**						
Sinaloa (Sin.)	*Oreochromis niloticus*	302	20	*Gyrodactylus cichlidarum*	KY489681	11399‡
					KY489682	11399‡
					KY489677	11400‡
				*Gyrodactylus yacatli*	–	11426**
Sonora (Son.)	*Oreochromis niloticus*	121	33	*Gyrodactylus cichlidarum*	KY489672	11404‡
					KY489683	11403‡
					KY489694	11403‡
					KY489695	11405‡
					KY489696	11404‡
Jalisco (Jal.)	*O. niloticus*	147	27	*Gyrodactylus cichlidarum*	KY489678	11391‡
					KY489680	11391‡
					KY489676	11393‡
					KY489679	11393‡
					KY489671	11396‡
					KY489675	11396‡
					KY489690	11397‡
				*Gyrodactylus yacatli*	KY489744	11420**
					KY489755	11420**
					KY489757	11420**
					KY489753	11420**
					KY489754	11420**
**3. East**						
Yucatán (Yuc.)	*Oreochromis niloticus*	43	37	*Gyrodactylus cichlidarum*	KY489668	–
					KY489669	–
					KY489670	–
					KY489673	–
					KY489674	–

*Oreochromis* PARGO-UNAM is a variety of 25% Rocky mountain (hybrid of *O. aureus* Steindachner x *O. niloticus*) and 25% red variant *O. niloticus* and 50% Florida red tilapia (hybrid of *O. mossambicus* x *O. urolepis hornorum* Trewavas). Rocky mountain (hybrid of *O. aureus* Steindachner x *O. niloticus*). Florida red tilapia (hybrid of *O. mossambicus* x *O. urolepis hornorum* Trewavas). CNHE ˗ Colección Nacional de Helmintos (Universidad Nacional Autónoma de México).

*Holotype, †Paratypes, **Hologenophore, ‡Vouchers.

Overall, 932 “tilapia” and native cichlids were inspected and 2173 gyrodactylids were recovered. Of these, a subsample of 285 parasites was analysed morphometrically and molecularly. No “spillover” parasites were found on any native fish species, other than native cichlids. Parasite abundance calculated for *Gyrodactylus* sp. (i.e., individuals that were not identified to species) ranged from 0.15 ± 0.15 worms/host ± S.D. for Yucatán to 17.9 ± 4.73 worms/host in Chiapas. Regionally, the East had the lowest mean parasite abundance (0.15 ± 0.15 worms/host), followed by the North-West (1.84 ± 2.74 worms/host) and the Centre-South (4.46 ± 5.01 worms/host); regions differed significantly in parasite abundance (Kruskall-Wallis, p < 0.001), with similarly high parasite abundances in the Centre-South and North-West (Mann–Whitney test, p = 0.2075), and significantly lower abundance in the East (Mann–Whitney test, p < 0.001).

Of the 285 parasites identified taxonomically using morphological and molecular characters, 254 worms were collected from “tilapia”, and 31 from native cichlids. Three species of *Gyrodactylus* infecting cichlid fishes in Mexico were identified, two previously described and one new species: *G. cichlidarum* (n = 236 worms; 43 ITS sequences obtained), *G. yacatli* (n = 44; 24 sequences), and *G. shinni* n. sp. (n = 5; 2 sequences) (Table 1). In addition, five *Gyrodactylus* specimens collected from *O. niloticus* cultured in Kenya were obtained and characterized. A single sequence was obtained from the Kenyan samples, corresponding to *G. yacatli*; the remaining four specimens were identified by morphology alone and confirmed to be *G. yacatli*.

### Nomenclatural acts

This published work and the nomenclatural acts it contains have been registered in ZooBank, the online registration system for the ICZN. The ZooBank Life Science Identifiers (LSIDs) can be resolved and the associated information viewed through any standard web browser by appending the LSID to the prefix “http:/zoobank.org/”. The LSID for this publication is: urn:lsid:zoobank.org:pub:166AB775-AA03-45A2-8F17-9AD33DB62A01. In addition, species profile including taxonomic traits, host details, and other metadata are provided on www.gyrodb.net^20,21^.

### *Gyrodactylus cichlidarum* (Fig. 2)

urn:lsid:zoobank.org:act:18730161-3470-4E74-BEC4-29D3F526E1DA

*Gyrodactylus cichlidarum* was the most abundant gyrodactylid infecting farmed “tilapia” in this survey—it was recorded on every farm sampled, and on all “tilapia” varieties inspected. It was most abundant in the Centre-South, and least abundant in the East of the country. This African parasite was found to infect three native cichlid fishes in Mexico: *P. nebuliferus* and *V. fenestrata* (both in Oaxaca State) and an unidentified native cichlid collected in Chiapas (Fig. 1, Table 1)—these constitute new host and locality records for this parasite. Overall, marginal hook morphology in most localities sampled was similar to that of specimens previously recorded^22^. However, slight differences in the size of the hooks were registered when contrasting regions and hosts. For instance, marginal hooks were between 21 and 28 µm in total length in the North-West and the Centre-South, compared with < 20.5 µm in the East. Parasite haptoral hard parts also varied in size depending on their host, as illustrated in a single farm in the state of Veracruz, where different “tilapia” strains are cultured. The hamuli of the gyrodactylids infecting “Rocky Mountain”, “Pargo UNAM”, and wild-type and red varieties of *O. niloticus* ranged considerably in size, from approx. 22 to 52 µm. On native cichlids collected in Oaxaca, the hamuli and marginal hooks of the parasites were larger compared with those obtained from cultured tilapia: in *P. nebuliferus* these were 66.7 and 29.8 µm long, respectively; followed by *V. fenestrata* with 65.2 and 29.7 µm long, respectively.

**Figure 2:**
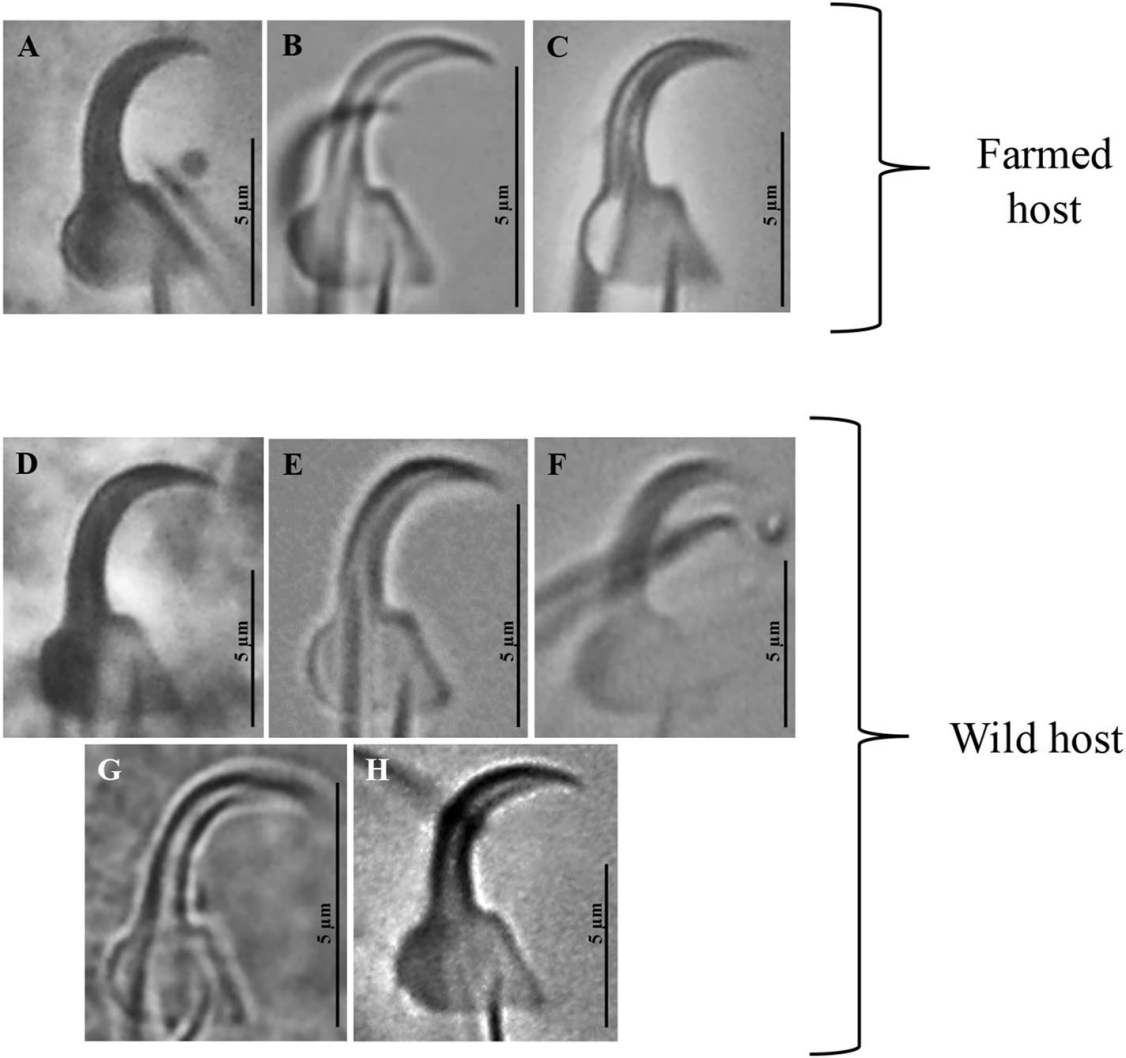
Micrographs of the marginal hook sickles of *Gyrodactylus cichlidarum* found infecting farmed “tilapia” and different native cichlids and poeciliids in Mexico. (**A**) *Oreochromis niloticus*, from Sonora. (**B**) *O. niloticus*, Yucatán. (**C**) *O. niloticus*, Chiapas. (**D**) *Paraneetroplus nebuliferus*. (**E**) *Vieja fenestrata*. (**F**) *Poecilia mexicana*. (**G**) *Poeciliopsis gracilis*. (H) *Pseudoxiphophorus bimaculatus*. Image H reprinted from Veterinary Parasitology, Vol 235, García-Vásquez A, Razo-Mendivil U & Rubio-Godoy M, Triple trouble? Invasive poeciliid fishes carry the tilapia pathogen *Gyrodactylus cichlidarum* (Paperna, 1968) in the Mexican highlands, Pages No. 37–40., Copyright (2017), with permission from Elsevier.

### Taxonomy

#### ***Gyrodactylus shinni*** n. sp. (Fig. 3; Tables 1, 2) n = 5

urn:lsid:zoobank.org:act:0DD8A6E3-7D9F-4AAB-8E48-A569764A887B

**Figure 3:**
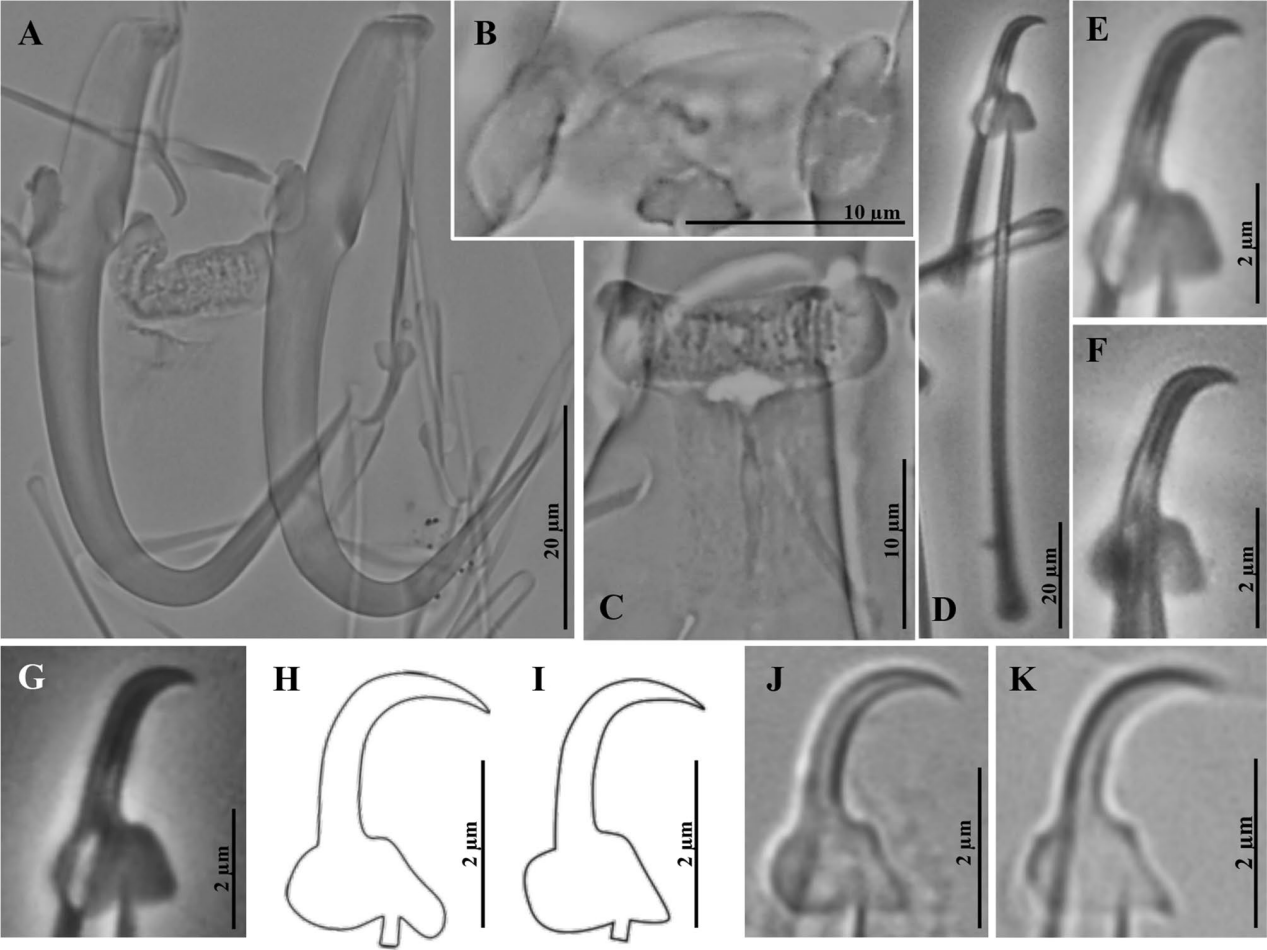
Micrographs of *Gyrodactylus shinni* n. sp. described from *Oreochromis niloticus* from Chiapas, Mexico; and comparison of marginal hook sickles to those of *Gyrodactylus* species found in cichlids with similar morphologies. (**A**) Hamuli complex. (**B**) Dorsal bar. (**C**) Ventral bar. (**D**) Marginal hook at a glance. (**E**,**F**) Marginal hook sickle. (**G**) Marginal hook sickles of *G. shinni* n. sp. compared with those of: (**H**) *G. occupatus* Zahrandníčková, Barson, Luus-Powell and Přikrylová, 2016 (re-examination; new drawing based on microphotograph kindly provided by Dr. Ivá Přikrylová). (**I**) *G. parisellei* Zahrandníčková, Barson, Luus-Powell and Přikrylová, 2016 (re-examination; new drawing based on microphotograph kindly provided by Dr. Ivá Přikrylová). (**J**) *G. ulinganisus* García-Vásquez, Hansen, Christison, Bron and Shinn, 2011. (**K**) *G. cichlidarum* (García-Vásquez, Hansen and Shinn, 2007).

Morphological description based on five proteolytically partially digested specimens. Hamuli slim 51.9 µm (50.8–52.7) long, 3.6 (3.2–3.9) wide, same width in all their length; shaft 33.5 (32.7–34.9) long; slender point 25.2 (24.5–25.9) long, comprising more than half of the shaft length; proximal shaft width 7.3 (7.3–7.8) wide; straight shaft; wide hamulus aperture distance 22.4 (21.4–24.2) long; broad hamulus aperture angle 44.2° (42.2°–48.2°), outlining “V”-like shape in the hamulus; hamulus root 20.4 (19.1–21.6) long, straight with rectangular edges, ventral side (facing host) ending in slight protuberance (Fig. 3a). Dorsal bar 20.8 (18.4–22.8) wide, 1.8 (1.4–2.1) long and curved, uniformly wide but becoming bulky at attachment point; long, oval attachment points, 7.4 (6.8–8.1) long (Fig. 3b). Ventral bar 19.3 (18.7–19.9) wide, 20.5 (17.7–21.6) long; very short ventral bar processes 1.4 (1.2–1.6) long, semi-rounded; ventral bar median portion 6.1 (5.2–6.6) long, slightly kidney-shaped with rounded edges; ventral bar membrane 13.3 (11.8–14.3) long, triangular (Fig. 3c). Marginal hook 40.0 (39.2–40.7) long; shaft long and slender, 32.5 (30.9–35.5) long. Marginal hook sickle 8.2 (8.1–8.3) long, shaft slightly tilted forwards, continuing into thick curvature ending in blunt point, almost reaching toe level; marginal hook distal width 4.1 (4.0–4.3); toe 1.8 (1.6–1.8) long, angular, curved bridge with trapezoidal shape ending in rounded point facing downwards (Fig. 3d–f). Marginal hook aperture 7.9 (7.7–8.0) 1ong. Heel rounded and prominent, level with toe bridge. Marginal hook instep height 0.4 (0.3–0.4) long, shallow. Filament loop 17.3 (16.5–18.5) long, a third of marginal hook total length (Fig. 3d).

**Table 2:** Morphological measurements of *Gyrodactylus shinni* n. sp. from *Oreochromis niloticus* (L.) and an unidentified native cichlid collected from Chiapas.

Measurement	*G. cichlidarum* Holotype	*Gyrodactylus shinni* n. sp. n = 5	*G. occupatus* n = 14*	*G. parisellei* n = 11*	*G. ulinganisus* n = 9*
HTL	54.3	50.8–52.7 (51.9)	66.1–73.8 (69.4)	48.5–53.5 (52.1)	59–65 (61.9)
HA	22.2	21.4–24.1 (22.4)	–	–	22–26 (23.3)
HPSW	7.7	7.3–7.9 (7.6)	–	–	7–9 (8.0)
HPL	24.3	24.5–25.9 (25.2)	26.8–33.4 (29.8)	19.8–23.5 (22.1)	27–30 (28.5)
HDSW	3.6	3.2–3.8 (3.5)	–	–	3–5 (4.1)
HSL	29.2	32.7–34.9 (33.5)	45.0–49.2 (46.9)	35.5–38.8 (37.6)	35–40 (37.6)
HICL	3.1	0.5–1.6 (1.1)	–	–	1–3 (1.8)
HAA°	45.2	42.1–48.2 (44.2)	–	–	36–48 (41.1)
HPCA°	11.4	1.5–7.6 (4.2)	–	–	4–8 (5.5)
IHAA°	51.5	48.3–54.0 (50.3)	–	–	41–50 (46.7)
HRL	21.4	19.1–21.5 (20.3)	24.7–29.8 (27.0)	16.2–19.9 (18.6)	22–28 (24.6)
VBL	19.2	17.6–21.5 (20.5)	21.5–24.1 (22.6)	17.0–21.2 (18.5)	23–26 (24.4)
VBW	19.6	18.7–19.9 (19.3)	7.7–13.3 (8.7)	8.9–10.6 (9.5)	22–27 (24.7)
VBPML	1.2	1.2–2.3 (1.9)	–	–	1–4 (2.5)
VBML	5.4	5.1–6.6 (6.0)	14.7–21.2 (17.2)	11.7–13.8 (12.3)	5–9 (7.6)
VBPL	1.5	1.2–1.5 (1.4)	–	–	1.5–3 (2.0)
VBMemL	12.9	11.8–14.3 (13.3)	–	–	18–24 (20.3)
DBL	1.1	1.3–2.1 (1.7)	1.2–2.3 (1.7)	1.2–1.8 (1.5)	2–2.5 (2.1)
DBW	19.0	18.4–22.8 (20.8)	15.7–19.3 (16.8)	13.4–15.8 (14.2)	12–16 (14.8)
DBAPTL	–	6.7–8.0 (7.4)	–	–	–
MHTL	27.9	39.2–40.7 (39.9)	31.6–37.9 (34.0)	27.8–32.5 (30.0)	28–32 (31.3)
MHSL	21.6	30.8–35.5 (32.4)	23.8–28 (26.3)	20.5–24.8 (22.7)	21–24 (23.7)
MHSiL	6.5	8.0–8.2 (8.1)	7.6–8.1 (7.8)	7.3–7.8 (7.6)	7–8 (7.7)
MHSiPW	2.9	3.9–4.2 (4.1)	4.2–4.9 (4.6)	3.6–4.7 (4.1)	4–5 (4.4)
MHToeL	1.1	1.6–1.8 (1.7)	–	–	1–2 (1.4)
MHSiDW	3.9	2.8–3.9 (3.4)	3.8–4.9 (4.6)	3.4–4.7 (3.9)	4.5–5.5 (4.7)
MHA	6.8	7.7–8.0 (7.8)	6.4–7.6 (7.4)	6.4–7.4 (7.6)	7–7.5 (7.1)
MHI/AH	0.2	0.2–0.4 (0.3)	–	–	0.1–0.3 (0.3)
MHFL	–	16.5–18.4 (17.2)	–	–	–

Data are presented alongside those of *G. cichlidarum* Paperna, 1968 from *Sarotherodon galilaeus* (L.) from Ghana, *G. occupatus* Zahradníčková, Barson, Luus-Powell & Přikrylová, 2016 from *O. niloticus* (L.) from Zimbabwe, *G. parisellei* Zahradníčková, Barson, Luus-Powell & Přikrylová, 2016 from *Pseudocrenilabrus philander* (Weber) which was collected from Zimbabwe, and *G. ulinganisus* García-Vásquez, Hansen, Christison, Bron & Shinn, 2011 from *O. niloticus* from South Africa. For each variable, the range, and the mean between parentheses are presented in micrometers.

*HTL* hamulus total length; *HA* Hamulus aperture; *HPSW* hamulus point shaft width; HPL Hamulus point length; *HDSW* Hamulus distal shaft width; *HSL* Hamulus shaft length; *HICL* Hamulus inner curve length; *HAA°* Hamulus aperture angle; *HPCA°* Hamulus point curve angle; *IHAA°* Inner hamulus aperture angle; *HRL* Hamulus root length; *VBL* Ventral bar length; *VBW* Ventral bar width; *VBPML* Ventral bar process to mid length; *VBML* Ventral bar median length; *VBPL* Ventral bar process length; *VBMemL* Ventral bar membrane length, *DBL* Dorsal bar length; *DBW* Dorsal bar width; *DBAPTL* Dorsal bar attachment point length; *MHTL* Marginal hook total length; *MHSL* Marginal hook shaft length; *MHSiL* Marginal hook sickle length; *MHSiPW* Marginal hook sickle point width; *MHToeL* Marginal hook toe length; *MHSiDW* Marginal hook sickle distal width; *MHA* Marginal hook aperture; *MHAA* Marginal hook aperture angle; *MHI/AH* Marginal hook instep / arch height; *MHFL* marginal hook filament loop. *measurements taken from the original descriptions (*G. occupatus* and *G. parisellei* Zahrandníčková et al., 2016; and *G. ulinganisus* García-Vásquez et al., 2011).

### Taxonomy summary

*Type host*: *Oreochromis niloticus.*

*Other host:* unidentified native cichlid.

*Site of infection*: Fins.

*Type locality*: Regal Springs tilapia farm, Chiapas State (17.449167°; -93.4475°).

*Collectors:* Adriana García and Miguel Rubio.

*Type specimens*: Holotype (accession no. CNHE 11387), paratypes (accession no. CNHE 11388) deposited in Colección Nacional de Helmintos (CNHE), Universidad Nacional Autónoma de México, Mexico City.

*Molecular data:* ITS rDNA sequences of *Gyrodactylus shinni* n. sp. (863 bp) obtained from two individuals collected from *O. niloticus* deposited in GenBank (Accession nos MN759066 - MN759067)*.* Lengths of the 18S, ITS1, 5.8S, ITS2 and 28S were 47, 342, 157, 303 and 20 bp, respectively.

*Etymology:* the species is named after Dr. Andrew Paul Shinn, for his contribution and passion for the taxonomy, systematics and general knowledge of *Gyrodactylus* species worldwide.

*Remarks*: Among hundreds of worms analysed, we only identified five specimens of this new species, *G. shinni*; and all of these specimens had been previously dissected to digest their haptors for morphological analysis, and their bodies used for molecular analysis. Thus, we cannot provide a whole body type specimen nor morphometric and other details on complete specimens. Nonetheless, the description of *G. shinni* n. sp. presented complies with the provisions of both the International Commission on Zoological Nomenclature (ICZN) Code and the World Organisation for Animal Health (OIE) Manual. The morphology of the haptoral hard parts of *G. shinni* n. sp. is very close to those of *G. cichlidarum*^23^, *G. ulinganisus*^24^*, G. occupatus*^18^ and *G. parisellei*^18^ (Fig. 3), the last three species being described from native cichlids in Africa. Despite their overall similarity, these species can be easily discriminated from *G. shinni* n. sp. by their marginal hook morphology. The marginal hook sickle shaft of all African species is straight and forms a deep, elongated closed curve reaching a relatively long point, while in *G. shinni* n. sp. the shaft tilts forward and ends in a comparatively short point (Figs. 3g-j). *Gyrodactylus shinni* n. sp. was recorded infecting farmed “tilapia” as well as an unidentified, native cichlid in Chiapas.

#### **Gyrodactylus yacatli **(Figs. 4, S1; Tables 1, 3)

urn:lsid:zoobank.org:act:C56E6FBA-AD86-4827-8161-143663D4A31D

Morphological revised description based on 44 proteolytically digested specimens, collected in the states of Chiapas (n = 5), Jalisco (n = 5), Sinaloa (n = 1), Oaxaca (n = 19) and Veracruz (n = 14), from different *O. niloticus* strains (n = 39 fish), from two species of native cichlids (*Vieja fenestrata* (n = 3), and an unidentified native cichlid (n = 1), and five specimens from cultured *O. niloticus* obtained from Kenya (Tables 1 and 3). Morphology of the haptoral hard parts of the new specimens studied is almost identical to original description. Ventral bar and marginal hooks presented some slight differences. Measurements used in the revised description are the mean from the five states where the species was collected, but separate measurements from each state are presented in Table 3, as these varied considerably between localities and fish host strains/species.

**Figure 4:**
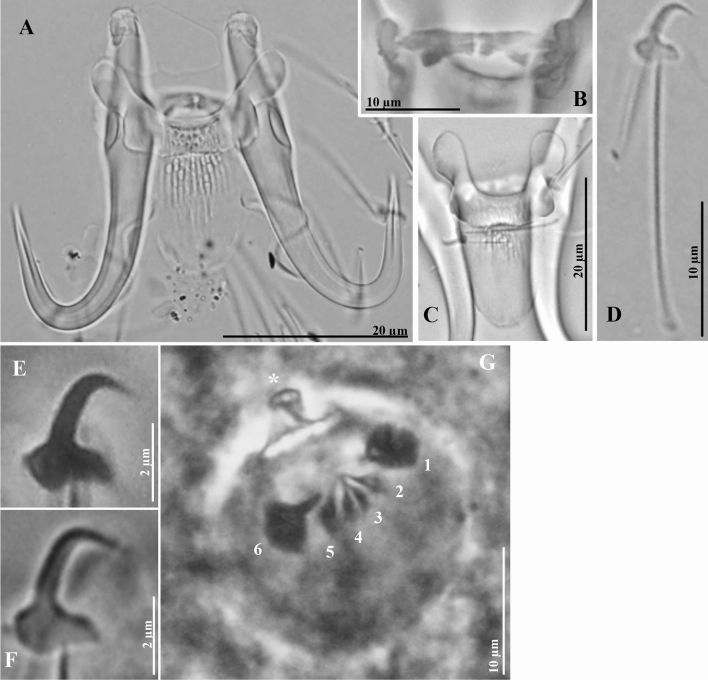
Micrographs of *Gyrodactylus yacatli* collected from Chiapas, Jalisco, Sinaloa, Oaxaca and Veracruz. (**A**) Hamuli complex. (**B**) Dorsal bar. (**C**) Ventral bar. (**D**) Marginal hook at a glance. (**E**,**F**) Marginal hook sickle. (**G**) Male Copulatory Organ (MCO) (star denotes principal hook and numbers denote the spines).

**Table 3:** Measurements of *Gyrodactylus yacatli* García-Vásquez, Hansen, Christison, Bron & Shinn, 2011 compared with specimens found in the Mexican states of Chiapas, Jalisco, Oaxaca and Veracruz infecting cultured “tilapias” and native cichlids, and specimens from cultured *Oreochromis niloticus* from Kenya, Africa.

Measurement	Original description n = 4*	Chiapas n = 4	Chiapas (Native cichlid) n = 1	Jalisco n = 5	Oaxaca n = 16	Oaxaca (*Vieja fenestrata*) n = 3	Sinaloa n = 1	Veracruz n = 14	Kenya n = 5
HTL	47–49 (48.4)	48.7–50.2 (49.6)	73.2	68.2–75.5 (24.6)	40.3–57.8 (44.3)	56.9–57.8 (57.4)	49.1	46.7–72.9 (51.7)	51.9–52.1 (52.0)
HA	15–18 (16.8)	15.2–18.6 (16.4)	22.3	23.3–25.7 (24.6)	10.8–18.8 (14.4)	18.3–18.8 (18.5)	17.7	14.8–25.3 (17.2)	15.2–17.8 (16.6)
HPSW	7–8 (7.9)	7.3–8.3 (7.7)	12.0	10.2–13.2 (11.7)	6.2–9.7 (7.6)	9.1–9.7 (9.4)	7.9	7.12–10.8 (8.4)	8.0–9.1 (8.4)
HPL	22–23 (22.7)	22.1–23.3 (22.8)	30.5	30.4–34.5 (33.1)	18.4–27.9 (20.6)	26.9–27.9 (27.4)	23.1	22.9–33.5 (23.9)	23.8–24.9 (24.5)
HDSW	3–4 (3.6)	3.3–3.8 (3.6)	5.3	4.8–5.6 (5.3)	2.9–4.4 (3.4)	2.3–4.4 (4.3)	3.7	3.1–5.4 (3.7)	3.7–4.5 (4.2)
HSL	31–33 (32.2)	32.1–32.5 (32.3)	49.9	45.0–49.2 (47.6)	26–37.7 (28.5)	36.8–37.8 (37.3)	30.8	30.9–45.7 (33.8)	31.5–33.9 (33.0)
HICL	1–2 (1.6)	0.6–1.1 (0.9)	0.9	0.4–1.4 (0.8)	0.4–1.2 (0.7)	1.0–1.2 (1.1)	0.9	0.5–1.5 (0.9)	0.8–1.3 (1.1)
HAA°	34–40 (37.9)	32.0–41.2 (36.1)	31.0	34.1–36.9 (35.6)	26.5–37.9 (33.7)	32.6–34.3 (33.5)	37.6	31.0–38.5 (35.4)	26.3–36.4 (32.2)
HPCA°	5–7 (6.2)	2.1–4.4 (3.1)	3.2	2.3–4.0 (3.0)	1.7–4.4 (3.3)	3.2–3.4 (3.3)	3.0	1.7–5.3 (3.2)	2.5–4.9 (3.9)
IHAA°	39–46 (43.3)	37.8–48.5 (42.4)	38.1	39.1–43.2 (41.5)	31.1–53.3 (40.7)	38.1–40.0 (39.0)	44.1	36.1–45.1 (41.1)	32.1–43.5 (38.1)
HRL	16–18 (16.9)	16.9–18.9 (17.9)	24.5	20.0–28.2 (24.3)	13.2–19.4 (15.3)	18.7–19.4 (19.1)	19.2	15.7–27.7 (18.2)	16.9–20.2 (18.7)
VBL	23–26 (24.7)	34.3–35.0 (34.7)	53.7	44.5–54.9 (57.8)	28.6–41.6 (33.8)	39.1–41.7 (40.4)	36.2	21.8–35.9 (29.3)	37.5–41.0 (39.8)
VBW	20–21 (20.4)	24.6–29.8 (27.1)	35.8	37.3–44.4 (40.9)	20.1–30.5 (24.3)	27.2–30.5 (28.9)	24.8	10.4–15.8 (11.7)	26.3–30.8 (27.8)
VBPML	11–12 (11.6)	9.9–12.3 (11.3)	16.9	13.7–18.2 (16.2)	9.2–14.4 (10.5)	13.6–14.4 (14.0)	12.1	9.4–17.1 (11.4)	13.1–13.7 (13.4)
VBML	5–6 (5.3)	5.1–5.8 (5.5)	6.9	6.7–8.4 (7.9)	4.1–6.9 (5.0)	5.8–6.9 (6.4)	5.5	4.6–7.7 (5.6)	5.2–8.2 (6.1)
VBPL	11–12 (11.5)	8.5–10.7 (9.4)	14	11.8–15.4 (13.7)	7.0–11.8 (8.9)	10.9–11.8 (11.3)	9.7	8.6–73.3 (13.3)	9.4–14.1 (11.1)
VBMemL	8–9 (8.5)	16.7–19.3 (18.0)	29.3	26.6–30.0 (28.4)	14.9–21.9 (16.8)	19.0–21.9 (20.5)	19.1	17.2–27.6 (20.2)	18.7–22.4 (20.7)
DBL	2.7	0.8–0.9 (0.8)	1.3	0.9–2.0 (1.5)	0.5–1.1 (0.9)	1.1–1.2 (1.1)	0.6	0.7–1.5 (1.1)	0.8–1.3 (1.0)
DBW	20.2	16.0–20.4 (18.1)	28.4	21.7–28.6 (26.0)	11.1–23.1 (16.6)	22.1–23.7 (22.9)	22.0	13.3–32.7 (18.1)	17.1–21.3 (19.0)
DBAPTL	–	6.2–7.3 (6.6)	9.5	8.3– 9.0 (9.9)	4.5–8.2 (5.7)	7.3–8.2 (7.8)	9.2	5.8–7.2 (6.5)	5.9–7.2 (6.5)
MHTL	22–24 (22.3)	22.2–23.6 (23.0)	32.5	27.2–28.9 (28.2)	18.9–29.1 (21.3)	28.3–29.1 (28.7)	21.6	19.51–31.8 (24.0)	22.3–23.9 (23.0)
MHSL	17–20 (18.0)	18.6–19.6 (18.8)	26.6	6.6–6.8 (6.7)	15.7–23.5 (17.6)	23.3–23.5 (23.4)	17.9	15.0–25.4 (19.4)	18.3–19.9 (18.9)
MHSiL	4–5 (4.5)	4.3–4.5 (4.4)	6.6	4.8–5.5 (5.2)	2.7–5.5 (3.9)	5.2–5.5 (5.4)	4.4	4.0–6.5 (4.6)	4.3–4.8 (4.6)
MHSiPW	3–4 (3.3)	3.1–3.6 (3.4)	5.3	2.2–2.7 (2.5)	1.4–4.2 (2.8)	3.8–4.2 (4.0)	3.7	2.8–5.2 (3.5)	3.2–3.8 (3.5)
MHToeL	1–2 (1.5)	1.3–1.7 (1.5)	2.4	4.1–4.6 (4.4)	1.1–2.1 (1.4)	1.8–2.1 (1.9)	1.5	1.2–2.2 (1.7)	1.4–1.7 (1.5)
MHSiDW	3–4 (3.2)	2.8–3.3 (3.1)	4	5.7–6.4 (6.0)	2.4–3.9 (2.9)	3.8–3.9 (3.8)	3.3	2.3–4.8 (3.1)	2.9–3.6 (3.3)
MHA	4–5 (4.2)	3.8–4.3 (4.0)	5.7	0.5–0.6 (0.6)	3.1–4.9 (3.6)	4.8–4.9 (4.8)	4.2	3.6–5.9 (4.2)	3.0–4.2 (3.9)
MHI/AH	0.3–0.4 (0.4)	0.2–0.4 (0.3)	0.5	14.1–16.7 (15.2)	0.2–0.6 (0.4)	0.4–0.6 (0.5)	0.3	0.3–0.6 (0.4)	0.4–0.8 (0.5)
MHFL	–	9.7–11.5 (10.9)	15.3	14.1–15.2 (16.7)	7.7–12.1 (9.4)	11.6–12.1 (11.8)	10.6	10.4–15.8 (11.7)	9.1–11.0 (9.9)

*HTL* hamulus total length; *HA* Hamulus aperture; *HPSW* hamulus point shaft width; HPL Hamulus point length; *HDSW* Hamulus distal shaft width; *HSL* Hamulus shaft length; *HICL* Hamulus inner curve length; *HAA°* Hamulus aperture angle; *HPCA°* Hamulus point curve angle; *IHAA°* Inner hamulus aperture angle; *HRL* Hamulus root length; *VBL* Ventral bar length; *VBW* Ventral bar width; *VBPML* Ventral bar process to mid length; *VBML* Ventral bar median length; *VBPL* Ventral bar process length; *VBMemL* Ventral bar membrane length, *DBL* Dorsal bar length; *DBW* Dorsal bar width; *DBAPTL* Dorsal bar attachment point length; *MHTL* Marginal hook total length; *MHSL* Marginal hook shaft length; *MHSiL* Marginal hook sickle length; *MHSiPW* Marginal hook sickle point width; *MHToeL* Marginal hook toe length; *MHSiDW* Marginal hook sickle distal width; *MHA* Marginal hook aperture; *MHAA* Marginal hook aperture angle; *MHI/AH* Marginal hook instep / arch height; *MHFL* marginal hook filament loop. *Measurements taken from the original description by García-Vásquez et al. 2011.

(Revised) Morphological description based on 50 specimens. Six whole worms cleared in ammonium picrate glycerine (APG), and 44 proteolytically digested. Body 33.1 (29.3–38.0) long; 10.4 (8.1–12.1) wide at uterus level. Pharynx 28.2 (25.9–29.7) long; 36.6 (29.2–43.6) width. Hamuli 51.9 µm (40.3–75.5) long, 5.6 (6.2–13.2) wide; shaft slightly curved 33.9 (26.0–49.9) long; slender point 24.0 (18.4–34.5) long, almost the same length as hamulus shaft; proximal shaft width 8.6 (6.2–13.2) wide, being the widest point of hamulus; hamulus aperture distance 17.2 (10.8–25.7) long; wide hamulus aperture angle 35.1° (30.4°–41.2°); hamulus root 18.1 (13.2–28.2) long, comprising one third of hamulus length, with semi-rounded edges, slightly narrower in its midsection (Fig. 4a). Dorsal bar 18.9 (11.1–32.7) wide, 1.0 (0.5–2.0) long, becoming narrow at attachment point, dorsal bar proper formed by two triangular sections with distinct junction in the middle, both tapering towards the middle, with a small mid-aperture; attachment points kidney-like, 6.6 (4.5–9.9) long, (Fig. 4b). Ventral bar 29.1 (20.1–44.4) wide, 37.7 (28.6–54.9) long; protuberant ventral bar processes 10.3 (7.0–15.4) long, narrower in mid-portion of bar proper and becoming rounded; ventral bar median portion 5.7 (4.1–8.4) long trapezoid shape, slightly curved basal section; ventral bar membrane 20.0 (14.9–30.0) long, more than half length of the hamulus shaft, lingulate shape (Fig. 4c). Marginal hook 24.5 (18.9–35.5) long; shaft long and slender, 20.1 (15.0–28.9) long (Fig. 4d). Marginal hook sickle 4.6 (2.7–6.8) long, proper sickle shaft angled forwards, forming long point ending beyond toe limit, slim point; marginal hook distal width 3.2 (2.3–4.8) wide; rhomboid toe 1.7 (1.1–2.7) long; slightly angled bridge, trapezoidal shape (Fig. 4e–f). Marginal hook aperture 4.2 (3.1–6.4) 1ong. Heel circular to rhomboid, ending below level of marginal sickle base. Marginal hook instep height 0.4 (0.2–0.6) long, forming curvature between heel and toe in attachment point of the marginal hook shaft. Filament loop 11.3 (7.7–16.7) long, almost half of the marginal hook shaft total length (Fig. 4d). Male copulatory organ (MCO) visible in three specimens only. Globular 18.5 (17.2–21.1) long, 19.5 (16.4–23.5) wide, consisting of one big principal hook 4.8 (4.0–5.9) long, two robust and four slim medium spines, all points facing each other towards centre of MCO; numbered clockwise from the principal hook: the two bigger spines (1 and 6) possess a wide base which becomes narrow developing a slim point; the other four spines (2–5): all positioned in front of the large spine, with points facing to middle, all with a very tight base and gradually narrowing into an acute point (Fig. 4g).

## Taxonomy summary

### Type host: *Oreochromis niloticus*

*Other hosts:* strains of “tilapia”: “Florida tilapia” (hybrid of *O. mossambicus* $\times$ *O. urolepis* (Norman)), “Rocky mountain” (hybrid of *O. aureus* Steindachner $\times$ *O. niloticus*), and “Pargo UNAM” (25% “Rocky mountain” + 25% red variant *O. niloticus* and 50% “Florida tilapia”), *Paraneetroplus nebuliferus*, *Vieja fenestrata* and an unidentified native cichlid.

*Site of infection*: Fins.

*Localities*: Regal Springs farm, Chiapas (17.449167°; − 93.4475°); Achitralada (19.742001°; − 104.126999°) and La Gigantera farms, Jalisco (20.567000°; -103.360001°); Río Chiquito, Cuicatlán, Oaxaca (17.811583; − 96.96075); Presa Picacho, Sinaloa (23.490258°; − 106.196167°); Jalacingo (19.636111°; − 96.421111°) and Rancho El Clarín, Tlapacoyan, Veracruz (20.035468°; − 97.106453°); and Sagana Aquaculture Centre, Kirinyaga County, Kenya (− 0.661111°; 37.2°).

*Collectors:* Adriana García, Miguel Rubio, Carlos Pinacho (Oaxaca), and Andrea Gustinelli (Kenya).

*Specimens*: Hologenophore specimens (Accession Nos. CNHE 11418, 11419, 11420, 11421, 11422, 11423, 11424, 11425 and 11426) deposited in Colección Nacional de Helmintos (CNHE), Universidad Nacional Autónoma de México, Mexico City.

*Molecular data:* ITS rDNA sequences of *Gyrodactylus yacatli* (975 bp) obtained from 25 individuals deposited in GenBank: Accession Nos. KY489740-42, MN759049 (Chiapas); KY489744, KY489753-55, KY489757 (Jalisco); MN759050-56 (Oaxaca); KY489745-50, KY489752, KY489756, MN759052 (Veracruz); and KY489739 (Kenya). Lengths of the 18S, ITS1, 5.8S, ITS2 and 28S were of 57, 358, 157, 384 and 19 bp, respectively.

**Figure 5:**
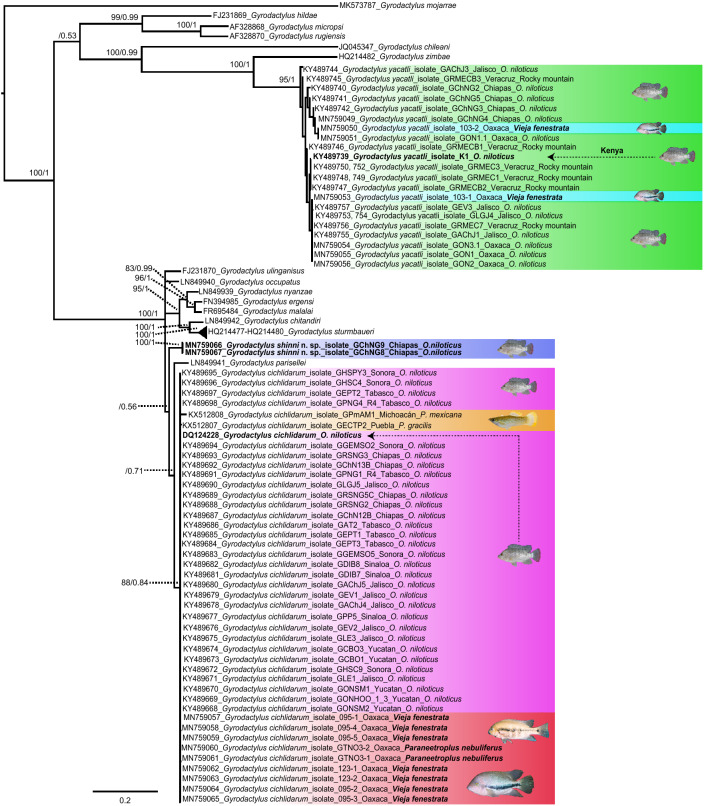
Phylogenetic hypothetical relationships for *Gyrodactylus* spp. collected from “tilapia” *Oreochromis niloticus* and from different native cichlids in Mexico. Phylogenetic trees inferred through Maximum Likelihood (ML) and Bayesian Inference (BI). Numbers near internal nodes show bootstrap and the posterior probability of clade frequencies. Scale bars indicate the number of substitutions per site.

*Remarks*: The species was originally described^24^ based on specimens collected in the states of Sinaloa, Tabasco and Yucatán, in Mexico. The description was based on morphology only, as no molecular data were obtained. Although overall the specimens used to describe *G. yacatli* and those collected in this study are very similar, slight morphological differences between them were detected, which we summarize below. In the description of the specimens from 2011, the ventral bar was found to have a squared membrane, but in most of the specimens analysed then, this structure was folded or broken making its description difficult. In the present study, we found that the membrane is long and lingulate (Fig. 4c). Morphologically, marginal hook sickles are very close to the original description, but their measurements vary considerably—probably reflecting intraspecific variation not discernible in the smaller and less geographically widespread sample described originally. Additionally, the specimens collected from native cichlids from Mexico (50.1 and 51.3 µm) and African (52.0 µm) farmed “tilapia” have longer hamuli than those of farmed *O. niloticus* in Mexico (44–51 µm). The hamulus aperture angle is wider in the specimens found in Sinaloa and Chiapas (37.6 and 35.1 µm, respectively) and narrower in Kenya and Oaxaca (33.0 and 33.7 µm, respectively). Marginal hooks in specimens collected from *V. fenestrata* are slightly larger than those collected from “tilapia” in Mexico and Kenya. In general, hook morphology of specimens found in the present study corresponds to those found in Kenya and with the original description (Fig. S1). Although no haptoral hook measurements are available for *G. yacatli* specimens recovered from Zimbabwe, marginal hook morphology from African and Mexican specimens generally agrees, with slight differences in the sickle shape: in Zimbabwean specimens, the toe is pointed downwards to the sickle base and makes a deep curvature where the sickle shaft is attached, whereas in the present study, the toe is rhomboid and the sickle base follows a slight square to rhomboid line. Also, the heel is rounded and the curvature in the inner marginal hook sickle is convex in Zahradníčková´s specimens (Fig. S1i), while in Mexican samples the heel is rhomboid and the inner marginal hook sickle is angled to triangular (Fig. S1g,h). In the present study, we were able to confirm that *G. yacatli* specimens found in Mexico and Kenya (Fig. S1j) are morphologically consistent; and provide an ITS sequence obtained from Kenyan samples that is almost identical to sequences generated from Mexican samples in this survey (Fig. 5). No molecular data are available from Zimbabwean *G. yacatli* specimens^18^, therefore, these cannot be compared phylogenetically to Mexican and Kenyan samples. New host and locality records for *G. yacatli* are provided in this study: the present is the first report of this species infecting native cichlid fishes in Mexico, including *P. nebuliferus*, *V. fenestrata*, and an unidentified cichlid species; new host records include species/hybrids of “tilapia”, such as “Rocky mountain”, “Florida tilapia” and “Pargo UNAM” (Table 1). The present study also constitutes the first locality record for some states in Mexico (Chiapas, Jalisco, Oaxaca and Veracruz; Table 1) and for Kenya on the African continent (Table 3).

### Genetic data

We obtained ITS sequences from gyrodactylids collected at 18 localities across Mexico and one from Kenya; this includes 43 sequences of *G. cichlidarum*, 24 of *G. yacatli* and two of *G. shinni* n. sp. The full ITS dataset (~ 913 pb) analysed included the 69 isolates generated in this study plus 15 nominal species of *Gyrodactylus* (i.e., *G. mojarrae*, *G. hildae*, *G. micropsi*, *G. rugiensis*, *G. chileani, G. zimbae, G. ulinganisus, G. occupatus, G. nyanzae, G. ergensi, G. malalai, G. chitandiri, G. sturmbaueri, G. parisellei*, and *G. cichlidarum* collected from native poeciliids in Mexico), with 646 variable sites for all samples. The sequence alignment of ITS showed numerous gaps of variable lengths (~ 1–86 bp). Nucleotide frequencies were A = 0. 242, C = 0. 217, G = 0. 233, and T = 0. 306. All GenBank accession numbers for newly generated sequences are provided in Table 1.

### Phylogenetic analyses

Phylogenetic trees inferred with ML and BI analyses yielded similar phylogenetic relationships. The ML tree had a value of − ln of 7452.44 (tree not shown). Both analyses showed that all sequences obtained in the current study corresponding to *G. cichlidarum*, *G. yacatli* and the new species described here, are nested in three independent clades with high bootstrap and posterior probability support values (Fig. 5). All isolates of *G. cichlidarum* from this study grouped with one sequence of *G. cichlidarum* available from GenBank (DQ124228) reported from *O. niloticus* in Scotland and with two sequences of this parasite collected from native poeciliids in Mexico (KX512807—KX512808); however, this clade showed low support values (88/0.84). *Gyrodactylus shinni* n. sp. formed a clade with high branch support (100/1); however, phylogenetic relationships were not clear (−/0.56). Finally, specimens of *G. yacatli* from Mexico and Kenya (KY489739) appear in a further clade with high branch support (95/1), grouped with *Gyrodactylus zimbae* infecting the cichlid *Simochromis diagramma* from Zambia (Fig. 5). Genetic divergence among the 43 new isolates of *G. cichlidarum* ranged from 0.1 to 0.3%, and with respect to *G. parisellei* variation was of 3.6%. Genetic distances between *G. shinni* n. sp., and the species *G. parisellei* and *G. cichlidarum* were 5.2% and 4.3%, respectively. Finally, the intra-specific variation of 24 isolates of *G. yacatli* ranged from 0.1 to 3.2%.

## Discussion

This survey provides evidence that three African gyrodactylid parasites translocated into Mexico with their “tilapia” hosts are now widely distributed in fish farms throughout the country; and have spilled-over to native cichlids. We cannot state when this happened (as “tilapias” were first introduced in 1945), nor can we suggest which fish species served as vector for the parasites (at least six different species of “tilapia” have been introduced over the years), and with the scant molecular evidence available, no inference/hypothesis can be made on what country in Africa was the original source of the parasites currently located in Mexico.

*Gyrodactylus cichlidarum* was initally described from Mango tilapia *Sarotherodon galilaeus* from Ghana, Africa^23^*.* This parasite has been recorded in several farmed and wild cichlids in Africa, including banded jewelfish *Hemichromis fasciatus* in Senegal^25^, Nile tilapia *O. niloticus* in Kenya^26^ and Mozambique tilapia *O. mossambicus* in Madagascar^27^; and outside Africa, it has been recorded on all continents except Antarctica^6,7,11^. *Gyrodactylus cichlidarum* is widely distributed in Mexico and infects not only farmed and feral “tilapia” throughout the country^12–17,28,29^, but has also been recorded on native poeciliid fishes^19^. This survey corroborates that this translocated African parasite is found throughout the country infecting several species/strains of “tilapia”, including *O. niloticus*, *O. mossambicus*, *O. aureus*, “Rocky mountain”, “Pargo UNAM”^30^ and “Florida tilapia”; and provides the first records of infection of three native cichlid fishes in Mexico: *P. nebuliferus* and *V. fenestrata* (both in Oaxaca) and an unidentified native cichlid collected in Chiapas. Thus, this study demonstrates that *G. cichlidarum* has quite low host specificity, and that although it exhibits marked intraspecific morphological variation when comparing individuals from different hosts and geographical locations, very limited molecular variation is present in this species.

When *G. yacatli* was originally described, the authors mentioned the possibility that this gyrodactylid species sporadically found in limited locations in Mexico “was an accidental infection or a host switch from a fish species inhabiting the water source feeding the farms, or probably has not yet been detected on the *O. niloticus* of African origin”^24^. In this study, we show that this parasite is widely distributed on both the Pacific and Atlantic sides of Mexico (with new host and locality records), and provide evidence that it represents a further translocated African species that is able to switch hosts to native, Neotropical cichlids. *Gyrodactylus yacatli* has previously been recorded in Zimbabwe and China^6,18^. We examined specimens from Zimbabwe and contrasted them with Mexican samples, and although minor morphological differences were found, specimens from both continents showed clear similarities. However, we cannot rule out that these are very similar/cryptic species, as no molecular data are available for the African samples. Chinese specimens^6^ could not be morphologically examined as mounted specimens could not be visualized properly; and no molecular data are available for these specimens either. Nonetheless, we were able to analyse *G. yacatli* specimens from Kenya using morphological and molecular characters, and these generally corresponded with those of Mexican samples. Phylogenetic analyses presented in this study show that the sample from Kenya sits within the clade containing all specimens found in Mexico. We consider these findings, and the fact that *G. yacatli* has been recorded in Zimbabwe and China, provide strong support for the notion that this is a further African gyrodactylid species translocated globally with the “tilapia” trade.

Recently, *G. mojarrae* was described as a species infecting several native cichlids from localities across southern Mexico (the Centre-South region in the present study), including Jack Dempsey *Rocio octofasciata*, chescla *Thorichthys maculipinnis*, Oaxaca cichlid *Vieja zonata* and *V. fenestrata*^31^. This species is clearly different morphologically from gyrodactylids found in African cichlids. Phylogenetic relationships among species of the genus *Gyrodactylus* associated with Neotropical and some African cichlids showed that *G*. *mojarrae* is clearly different from its congeners infecting “tilapia”^31^. The present study corroborates that finding, as *G*. *mojarrae* is sufficiently distinct from “tilapia” parasites to root the phylogenetic tree. In the phylogenetic hypothesis we present, the three gyrodactylid species found in this study do not show high intraspecific variation, and all are closely related to parasite species recorded from African cichlids –strengthening the idea that *G. cichlidarum*, *G*. *yacatli* and *G. shinni* n. sp. are translocated “tilapia” parasites of African origin.

In this survey, we inspected several non-cichlid native fishes collected in the Centre-South (the region with the highest abundance of “tilapia” gyrodactylids) and found no evidence of spillover. Furthermore, over the past decade we have studied gyrodactylids of Mexican native fishes and have not found evidence of parasite transfers from “tilapia” to other common fishes during studies on several species of *Gyrodactylus* infecting poeciliids, goodeids, profundulids, characids and cyprinids^24,32–39^. Thus, we are confident that other than the instances of parasite spillover from “tilapia” to native cichlids recorded here, and the rare instances of *G. cichlidarum* infecting poeciliids reported previously^19^, African gyrodactylids do not commonly switch host to non-cichlid fishes. As would be expected, farmed fish had higher infection parameters than wild fish, a situation probably resulting from both ecological conditions favouring parasite transmission among confined hosts, as well as a long co-evolutionary trajectory between *G. cichlidarum* and its African hosts. Although outside the remit of this survey, our findings generally coincide with the hypothesis suggesting that the major factors influencing the success of host switching are compatibility and opportunity^40^. Neotropical cichlids are a monophyletic sister clade related to African cichlids^41^, and thus are more compatible to their African parasites than more distantly related fishes; i.e., Neotropical cichlid fishes probably do not exert a sufficiently robust selective pressure to eliminate colonizing parasite populations. Significant intraspecific variation was observed in the size of haptoral hard parts when analyzing parasites collected from farmed “tilapia” and wild, native cichlid fishes, which probably reflects both phenotypic plasticity and adaptation to different hosts—and which may also contribute to increasing compatibility. Opportunity, i.e., the possibility of coinciding spatially and temporally with potential new hosts, was provided with the anthropogenic translocation of “tilapia” and their parasites; and further assisted by the generally lax management practices exerted in “tilapia” aquaculture in Mexico^14^, which facilitate both parasite growth within and their escape from fish farms.

Finally, we provide evidence that species of *Gyrodactylus* introduced with their “tilapia” hosts over the years are now widely distributed in Mexican fish farms and have spilled-over to native cichlid fishes, a situation that will most probably be replicated elsewhere in the Americas, considering “tilapias” are highly invasive fish species^4,42^ and monogeneans, including gyrodactylids, are known to be widely distributed in fish farms in the region^11^. Further studies are needed to assess the potential impact of translocated gyrodactylids on native fish hosts, considering monogenean parasites have been shown to be very successful invaders following co-introduction with their primary hosts^8,43–45^. This is of particular interest, taking into account that Monogenea have been shown to use a “stepping-stone” mode of host-switching enabling them to successfully colonize distantly-related Neotropical freshwater fish lineages^46^.

## Materials and methods

### Sample collection and preparation

During surveys conducted between 2013 and 2018, “tilapia” *Oreochromis niloticus* (several strains and hybrids) were collected from 40 farms located in three different, broadly defined regions in Mexico: North-West (States of Jalisco, Sinaloa and Sonora), Centre-South (States of Puebla, Oaxaca, Veracruz, Tabasco and Chiapas), and East (State of Yucatán) (see Fig. 1; Table 1). Randomly-selected, healthy fish were sacrificed with an overdose of anaesthetic, 2-phenoxyethanol (Sigma-Aldrich, Missouri) prior to storage in tubes containing 95% ethanol. A sample size 20–25 per farm was considered reasonably robust^47^. No control group was assessed, as this was a survey aimed at determining the distribution of parasites in the country, and eventually contrast findings between geographical regions, not within fish farms nor between experimental groups. A sample of gyrodactylid parasites collected from *O. niloticus* in Kenya was kindly donated for us to analyse by Dr. Andrea Gustinelli (University of Bologna, Italy) and Dr. Giuseppe Paladini (Stirling University, UK). To assess parasite spillover from “tilapia” farms, particularly in the Centre-South where several native cichlids occur, we also collected samples of common native fish families from streams and rivers close to farms, including species of Cichlidae, Poeciliidae, Goodeidae and Profundulidae. In the lab, ethanol-preserved fish were examined under a stereomicroscope (Zeigen model 60/666, Mexico City). Worms were removed with the use of surgical needles and were processed individually. Dislodged worms found in the bottom of the tubes where fish samples were kept were also recovered. The total number of worms found in samples from each farm was counted; and a subsample was taken for morphological and molecular identification. For species identification, haptors were excised using a scalpel and subjected to partial proteolytic digestion to remove tissue enclosing the haptoral armature, under the dissection microscope^48^. Digestion was arrested by the addition of a 50:50 glycerine/formalin solution (making a semi-permanent preparation), and specimens were then coverslipped and sealed with nail varnish. Bodies were fixed in 95% ethanol and stored at – 20 °C, individually labelled for subsequent molecular analyses.

### Morphological analysis

Partially digested haptoral hard parts were studied under a Leica DM 750 compound microscope (magnification of 10 × 100 with oil immersion lens for hamuli and the marginal hooks with negative phase contrast). Pictures were obtained using imaging analysis software Leica Application Suite, LAS ver. 4.12.0 with a Leica ICC50 HD camera. Using ImageJ 1.52a software, measurements were calculated from images. *Gyrodactylus* specimens found on cichlids (wild and cultured) and some native fish were compared with described species found worldwide^33^ (see Tables 2 and 3).

### Molecular data

The bodies of excised specimens whose haptors had been morphometrically characterized were placed individually in 1.5 ml microcentrifuge tubes for genomic DNA extraction using the DNeasy Blood & Tissue Kit (Qiagen, California) following manufacturer’s instructions. The ribosomal region spanning the 3′ end of the 18S rRNA gene, ITS1, 5.8S rRNA gene, ITS2, and the 5′ end of the 28S rRNA gene was amplified by PCR conditions^33^ using the primer pairs ITS1-fm (5'-TAGAGGAAGTACAAGTCG-3') and ITS2-rm (5'-GCTYGAATCGAGGTCAGGAC-3′)^33^. Additionally, the forward primer BD1, (5′-GTCGTAACAAGGTTTCCGTA-3′) and the reverse primer BD2, (5′-ATCTAGACCGGACTAGGCTGTG-3′)^49^ were used for some specimens. Amplicons were visualized on GelRed (Biotium, California) stained 1% agarose gel and then unincorporated nucleotides and primers of each PCR amplicon were removed using ExoSap-IT (USB Corporation, Ohio). Sequencing reactions were carried out with the use of BigDye Terminator v3.1 chemistry, incorporating the same primers as those used in PCR and internal primers ITSR3A (5′-GAGCCGAGTGATCCACC-3′) and ITS4.5 (5′-CATCGGTCTCTCGAACG-3′)^50^, and cleaned by filtration with Sephadex G-50. Sequenced products were read on an ABI PRISM 3100 automated DNA sequencer (Applied Biosystems, California). Electropherograms were visually inspected and overlapping fragments of forward and reverse sequences were assembled using Geneious 8.1.8^51^ (https://www.geneious.com). Sequences were deposited in GenBank and their accession numbers are cited in the description of each species.

### Alignment and phylogenetic analyses

Sequences of the ITS obtained in this study for the species described here, *Gyrodactylus shinni* n. sp*.* (2 worms MN759066, MN759067); *G. cichlidarum* (JN398477, KX512808, KX512807, KY489669-KY489698, MN759057-MN759065) and *G. yacatli* (KY489740-KY489757, MN759049-MN759056, KY489739 (Kenya)) were aligned with sequences of other *Gyrodactylus* spp. available in the GenBank database: *G. cichlidarum* (DQ124228); *G. mojarrae* (MK573785-87); *G. hildae* (FJ231869); *G. micropsi* (AF328868); *G. rugiensis* (AF328870); *G. chileani* (JQ045347); *G. zimbae* (HQ214482); *G. ulinganisus* (FJ231870); *G. occupatus* (LN849940); *G. nyanzae* (LN849939); *G. ergensi* (FN394985); *G. malalai* (FR695484); *G. chitandiri* (LN849942); *G. sturmbaueri* (HQ214477-HQ214480, LN849938); and *G. parisellei* (LN849941). Sequences were aligned using ClustalW with default parameters implemented in MEGA version 7.0^52^. The best-fitting nucleotide substitution model (GTR + G) was estimated with the Akaike Information Criterion (AIC) implemented in MEGA version 7.0. Phylogenetic trees were reconstructed by Maximum Likelihood (ML) and Bayesian inference (BI) analyses. For ML analyses, the program RAxML v7.0.4^53^ was used. A GTRGAMMAI substitution model was used for ML analyses, and 1000 bootstrap replicates were run to assess nodal support. BI trees were generated using MrBayes v3.2^54^, running two independent MC3 runs of four chains for 10 million generations and sampling tree topologies every 1000 generations. ‘Burn-in’ periods were set to 2.5 million generations according to the standard deviation of split frequencies values (p ˂ 0.01). Posterior probabilities of clades were obtained from 50% majority rule consensus of sample trees after excluding the initial 25% as ‘burn-in’. Genetic divergence among species of *Gyrodactylus* was estimated using uncorrected “*p*” distances in MEGA version 7.0^52^. Finally, trees were drawn using FigTree version 1.3.1^55^.

### General ecological and geographic patterns

Parasite abundance was calculated by dividing the total number of *Gyrodactylus* sp. worms found on farmed “tilapia” by the total number of fish examined in each farm and state surveyed (Table 1); i.e., no precise ecological data are available for individual gyrodactylid species, as not all parasite specimens were identified taxonomically and no ecological data are presented for *Gyrodactylus* sp. infections of native fishes. Significant numbers of parasites were dislodged from fish during transport, so we decided against presenting data on prevalence of infection (% of the sample infected). The proportion of parasite species reported for each state was calculated from the subsample of worms characterized morphologically and molecularly (Table 1). From this partial data (subsample), a summary map was constructed for visualization of this information using QGIS 3.6.2 software^56^.

To compare mean abundances of *Gyrodactylus* sp. between regions, data from farms in these regions was pooled (East = 14 farms, North-West = 14, Centre-South = 12), and the Kruskall-Wallis non-parametric test was performed to detect differences between these groups. Paired Mann–Whitney U tests were used to identify significantly different groups. Statistical tests were performed in R 3.6.0^57^.

### Ethics approval

The authors declare that the work reported here was conducted following guidelines on the ethical treatment of animals and was authorized by the competent Mexican authorities: Ministries of Agriculture (SAGARPA) permit DGOPA/01173/120208.0107 and the Environment (SEMARNAT) permit SGPA/DGVS/02967/14.

## Supplementary Information

Supplementary Information 1.

Supplementary Information 2.

## Data Availability

The datasets generated during and/or analysed during the current study are available from the corresponding author on reasonable request.

## ARRIVE guidelines

This survey was carried out in compliance with the ARRIVE guidelines.
