# Supplementary figures and images for: Morpho-molecular characterization of Gyrodactylus parasites of farmed tilapia and their spillover to native fishes in Mexico
Source: Sci Rep. 2021 Jul 6;11:13957. doi: 10.1038/s41598-021-93472-6 (PMC8260806; doi:10.1038/s41598-021-93472-6)

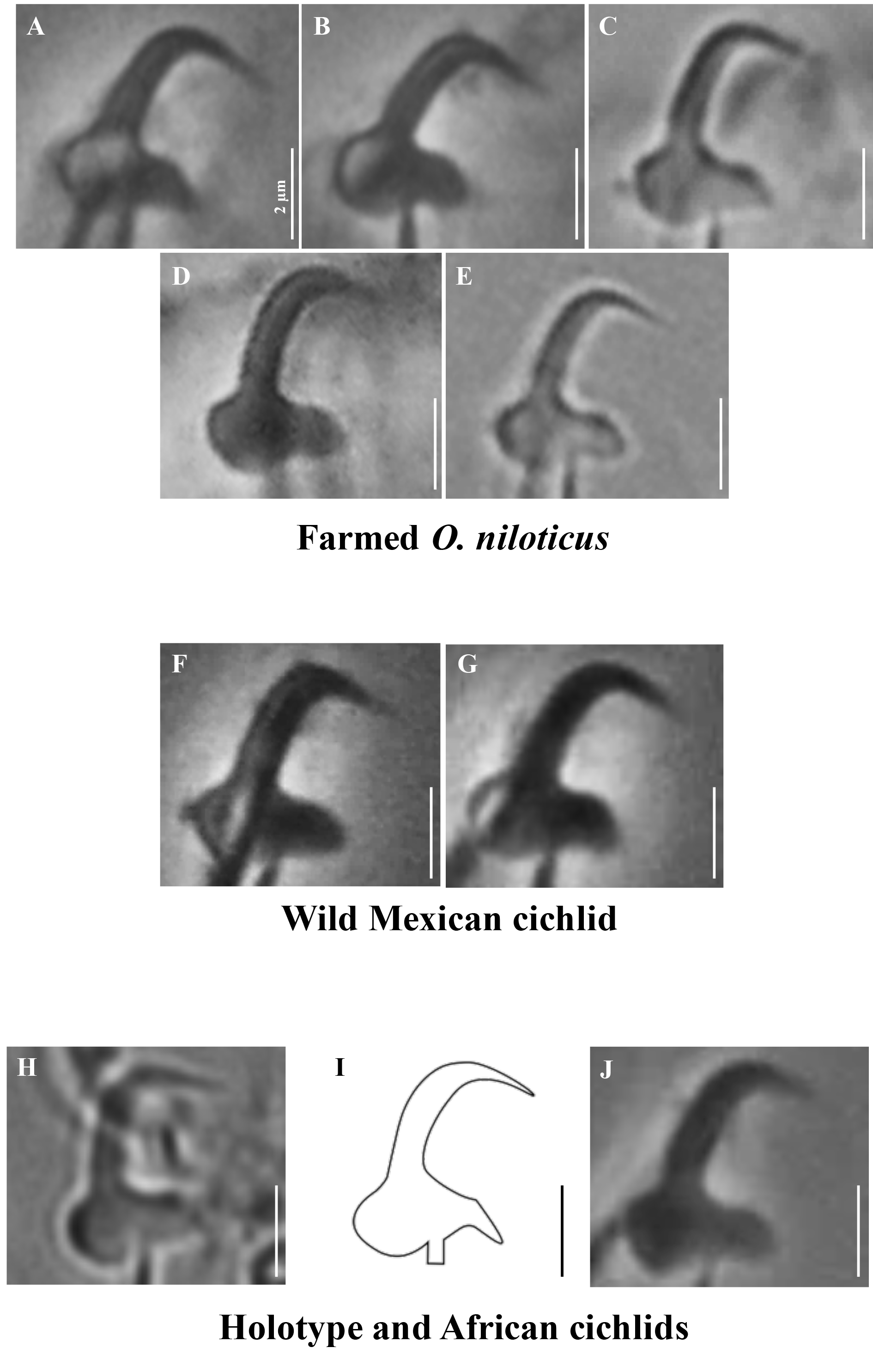

Supplement: Supplementary file 1 — Supplementary Information 1. [file 41598_2021_93472_MOESM1_ESM.tiff]
