# Supplementary material for: Morpho-molecular characterization of Gyrodactylus parasites of farmed tilapia and their spillover to native fishes in Mexico
Source: Sci Rep. 2021 Jul 6;11:13957. doi: 10.1038/s41598-021-93472-6 (PMC8260806; doi:10.1038/s41598-021-93472-6)
Supplement: Supplementary file 2 — Supplementary Information 2. [file 41598_2021_93472_MOESM2_ESM.docx]

**Legend to Figure S1**

**Figure S1.** Micrographs of the marginal hook sickles of *Gyrodactylus yacatli* found on farmed “tilapia” *Oreochromis niloticus* and some native cichlids in Mexico, compared with specimens previously recorded in Mexico and Africa. **(A-B)** *O. niloticus*, Oaxaca. **(C)** *O. niloticus* (“Rocky mountain” strain), Veracruz. **(D)** *O. niloticus*, Jalisco. **(E)** *O. niloticus*, Chiapas. **(F-G)** *Vieja fenestrata*, Oaxaca. **(H)** *G yacatli* from Mexico, collected in 2007 by Dr. Adriana García-Vásquez; previously unpublished, as this microphotograph was not included in original description by García-Vásquez et al. (2011). **(I)** Drawing of marginal hook sickle of *G. yacatli* from Zimbabwe, collected by Zahradníčková et al. (2016), new drawing based on microphotograph kindly provided by Dr. Ivá Přikrylová. **(J)** *G. yacatli* from Kenya.
